# Do PDGM Clinical Groups Disadvantage People With Dementia? A Pre-Implementation Baseline Analysis

**DOI:** 10.1093/geroni/igaf122.3747

**Published:** 2025-12-31

**Authors:** Maxwell Cutty, Carmen Pfeil, Sara Knox

**Affiliations:** Medical University of South Carolina, Charleston, South Carolina, United States; University of California, Los Angeles, Los Angeles, California, United States; Medical University of South Carolina, Charleston, South Carolina, United States

## Abstract

In January 2020, Medicare implemented the Patient-Driven Groupings Model (PDGM), linking 30-day home health payments to clinical (case-mix) groupings. How this affects individuals with Alzheimer’s Disease and Related Dementias (ADRD) is still not well understood. To establish a pre-PDGM baseline, we retrospectively applied PDGM rules to 2019 Medicare fee-for-service claims to simulate clinical grouping assignment for beneficiaries with and without ADRD (N = 1,432,374; 90% non-ADRD, 10% ADRD). We repeated this in a 1:1 propensity score-matched cohort stratified by ADRD status (129,428 per group), matching on demographics, comorbidity burden, prior health care use, social support, and baseline function/cognition. Before matching, ADRD patients were disproportionately represented in Neuro Rehab, while non-ADRD patients were concentrated in Surgical Aftercare. Notably, Behavioral Health, though small (0.94%), was the only clinical group with a majority of ADRD patients (65% vs. 3.1–17.7% in other groups). The top clinical groups in the stratified pre-match cohort were Musculoskeletal Rehabilitation (non-ADRD 34.0%; ADRD 20.1%) and Cardiac & Circulatory (non-ADRD 16.6%; ADRD 21.1%). After matching, most clinical groups converged toward a 50/50 mix of non-ADRD/ADRD patients. However, Behavioral Health and Neuro Rehab skewed toward ADRD (92.1%, 59.3%), while Infectious Disease, Surgical Aftercare, and Respiratory skewed toward non-ADRD (64.0%, 64.1%, 59.3%). These findings provide the first pre-PDGM characterization of ADRD patient distribution across clinical groupings, highlighting how reimbursement incentives may differentially affect beneficiaries with ADRD. This timely evidence establishes a foundation for evaluating whether the PDGM inadvertently disadvantages patients with ADRD, informing policy, clinical practice, and future research on equitable home health delivery.

